# Therapeutic Efficacy of Autologous Blood-Derived Stem Cells with Growth Factors in Moderate to Severe Alzheimer’s Disease: A Clinical Trial

**DOI:** 10.1007/s12035-025-05583-0

**Published:** 2025-12-10

**Authors:** Woong Jin Lee, Kyoungjoo Cho, Dayoon Lee, Seungmin Lee, Gyung Whan Kim

**Affiliations:** 1 Department of Neurology, College of Medicine, Yonsei University Health System, Seoul, South Korea; 2 Department of Life Science, Kyonggi University, Suwon, South Korea; 3 College of Medicine, Ewha Womans University, Seoul, South Korea

**Keywords:** Alzheimer’s disease, Autologous blood-derived stem cells, Clinical trial, SMART M-CELL2

## Abstract

Alzheimer’s disease (AD) is characterized by cognitive decline, memory loss, and a gradual loss of daily functioning. Unfortunately, despite extensive research, effective treatments for AD remain limited. Of these, stem cell-based therapies show promise for their regenerative potential and ability to modulate pathological processes. Autologous blood-derived stem cells (ABSCs), which are isolated from a patient’s own blood, have demonstrated therapeutic efficacy in AD. This clinical study evaluated the safety and efficacy of ABSCs on patients with AD and investigated the changing levels of growth factors derived from ABSCs treatment. The efficacy of the treatment on cognitive function was assessed using the Mini-Mental State Examination, Clinical Dementia Rating, and AD Assessment Scale-Cognitive Subscale, all widely used tools to assess cognitive function in patients with AD. The neuroimaging and molecular mechanisms were the secondary outcomes. The neuroimaging examinations performed included PET-CT with amyloid imaging, for assessing amyloid plaque deposition in the brain at baseline and at 3 and 6 months after treatment; FDG-PET, for measuring brain glucose metabolism and acquiring insights into neuronal activity and overall brain function; and MRI, performed at baseline and follow-up, for assessing structural brain changes. ABSCs treatment resulted in notable improvements in cognitive function, reductions in amyloid plaque burden, and improved neuroimaging outcomes. Autologous stem cell therapy also reduced the risk of immune rejection, offering a safety advantage over allogeneic stem cell therapies. Furthermore, the use of growth factors to enhance stem cell efficacy aligns with existing research demonstrating improvements in stem cell limitations. This study provides compelling evidence that ABSCs combined with growth factors exhibit significant therapeutic potential for patients with moderate to severe AD. Our findings indicate that our current combination treatment may offer a multi-target approach to addressing the complex pathogenesis of neurodegenerative diseases and is thereby a potentially sustainable therapeutic strategy for AD. Furthermore, the combination of ABSCs with growth factors can potentially provide a much-needed therapeutic alternative for AD.

## Introduction

Alzheimer’s disease (AD) is a progressive neurodegenerative disorder and a prominently challenging public health concern worldwide [1]. It is characterized by cognitive decline, memory loss, and a gradual loss of daily functioning, which imposes a significant caregiver burden and societal costs. AD primarily affects individuals aged > 65 years, and its prevalence is expected to increase dramatically as populations age [2]. Despite extensive research, effective treatments for AD remain limited, and current therapeutic approaches, such as acetylcholinesterase inhibitors and glutamate regulators, only provide symptomatic relief without inhibiting disease progression [3]. As such, novel and more effective therapies are needed to target the underlying pathophysiology of AD, such as neuroinflammation, amyloid plaque deposition, and tau tangles.

Stem cell-based therapies have shown promise in the treatment of various neurodegenerative diseases, including AD, owing to their regenerative potential and ability to mitigate pathological processes [4]. In particular, autologous blood-derived stem cells (ABSCs), which are isolated from a patient’s own blood, have garnered increasing attention because of their lower risk of immune rejection, availability, and ease of isolation. Recent studies have demonstrated that ABSCs, especially when combined with growth factors and other bioactive molecules, can promote neuroprotection, enhance cellular regeneration, and modulate inflammatory responses, making them an attractive therapeutic option in AD [5]. However, scalability and cost-effectiveness are needed to broaden the clinical application of ABSC therapy. Autologous stem cells can be labor-intensive and costly to isolate and grow, which may limit their feasibility in resource-constrained settings. The development of more efficient methods for stem cell isolation, expansion, and delivery will be critical for making this treatment more accessible. Furthermore, as the use of stem cell therapies progresses toward routine clinical applications, potential long-term risks, including tumorigenesis or unwanted immune responses, must be continuously monitored.

The therapeutic efficacy of ABSCs in AD is supported by preclinical studies that have demonstrated their ability to improve cognitive function and reduce the amyloid burden in animal models [6]. These effects may be achieved through several mechanisms, such as neuronal regeneration, reduced neuroinflammation, and enhanced synaptic plasticity. Furthermore, growth factors, such as brain-derived neurotrophic factor (BDNF), vascular endothelial growth factor (VEGF), and insulin-like growth factor-1 (IGF-1), can promote stem cell survival, differentiation, and neuroprotection in neurodegenerative conditions [7]. However, clinical evidence supporting the use of ABSCs in human patients with AD is scarce, with only a few small-scale studies reporting promising results [8]. Furthermore, dysregulation of the PI3K/Akt and MAPK/ERK signaling pathways, which are crucial in cell survival, neuroprotection, and synaptic plasticity, has been implicated in the pathogenesis of AD and contributes to neuronal apoptosis, decreased neurogenesis, and impaired synaptic function. The PI3K/Akt pathway, which is activated by various growth factors, promotes cell survival and inhibits apoptosis by regulating downstream targets, such as mTOR and GSK3β. The reduced activation of this pathway in AD has been associated with increased tau phosphorylation, a hallmark of the disease. In comparison, the MAPK/ERK pathway is involved in synaptic plasticity, memory formation, and cell differentiation. The impairment of this pathway has been linked to cognitive decline in AD because of its key role in synaptic remodeling and neuroprotection [9]. Combining ABSCs with growth factors may activate these signaling pathways, thereby enhancing neuronal survival, reducing neuroinflammation, and improving cognitive function.

This clinical trial evaluated the safety, efficacy, and underlying mechanisms of the combined treatment of ABSCs and growth factors in patients with moderate to severe AD. We hypothesized that the combination of ABSCs with bioactive molecules will activate the PI3K/Akt and MAPK/ERK signaling pathways, improving neuronal survival, enhancing cognitive function, and mitigating disease progression. The study also explored the safety and feasibility of this combination therapy in a clinical setting by evaluating adverse events, immune responses, and other potential risks associated with stem cell therapy.

## Materials and Methods

### Study Design

This was a prospective single-site randomized controlled trial (RCT) that evaluated the therapeutic efficacy, safety, and mechanistic insights into the effects of the combined treatment of ABSCs combined with growth factors in patients with moderate to severe AD [10]. The trial assessed clinical outcomes, including cognitive function, and explored the molecular mechanisms underlying the therapeutic effects, with a particular focus on the PI3K/Akt and MAPK/ERK signaling pathways. This study was approved by the institutional review board (IRB, 1–2023-0039) of Severance Hospital, and all patients provided written informed consent prior to participation (trial registration number, KCT0008278; date of registration, August 9, 2023). This study was designed as an exploratory pilot RCT; therefore, no formal priori power calculation was performed. The sample size was informed by effect sizes reported in previous early-phase cell-therapy studies. For this study, randomization was performed using a computer-generated block sequence (block size = 4), and allocation was concealed using sequentially numbered, sealed, opaque envelopes opened only after enrollment. Although participants and treating clinicians were not blinded, all cognitive assessors, PET/FDG imaging readers, and laboratory personnel were fully blinded to group assignment.

### Patient Recruitment and Eligibility Criteria

Eligible participants were adults aged 60–80 years diagnosed with moderate to severe AD (Clinical Dementia Rating–Sum of Boxes (CDR-SOB) ≥ 1; Mini-Mental State Examination (MMSE) score ≤ 24). The diagnosis was confirmed by clinical evaluation and supported by neuroimaging (e.g., amyloid positron emission tomography–computed tomography [PET-CT]). Patients were further required to meet the following inclusion criteria:

- Diagnosis of moderate to severe AD as per the National Institute on Aging–Alzheimer’s Association criteria [11];
- Positive beta-amyloid PET-CT results confirming amyloid plaque deposition [12];
- CDR-SOB ≥ 1 or MMSE score ≤ 24 [13]; and
- A stable medical condition without significant underlying diseases or conditions that could interfere with treatment.

The exclusion criteria were the following:

- History of severe adverse reactions to stem cell therapies or growth factors;
- Active cancer or other serious comorbidities;
- History of major neurological disorders other than AD (e.g., Parkinson’s disease, stroke); and
- Inability to provide informed consent or comply with the study procedures.

### Study Groups

Participants were randomly assigned to one of two groups:

#### Experimental Group (ABSCs + Growth Factors)

Patients in this group administered ABSCs combined with a cocktail of growth factors consisting of BDNF, VEGF, and IGF-1.

#### Control Group (Standard Care)

Patients in the control group underwent standard AD treatment (e.g., acetylcholinesterase inhibitors and memantine) but did not receive either ABSCs or growth factors.

### Stem Cell Isolation from the Blood of Patients and Processing

Autologous blood samples (220–260 mL of peripheral blood) were collected from all participants at the start of the study. Blood was processed using the SMART M-CELL2 system (Miracell, Seongnam, South Korea), a centrifugation device designed for stem cell isolation and concentration. The blood samples were subjected to density gradient centrifugation to separate blood components and isolate the peripheral blood mononuclear cells (PBMCs, 1.5–3.0 × 10^8^ mononuclear cells), which include stem cells, specifically mesenchymal stem cells (MSCs) [14]. Flow-cytometric immunophenotyping demonstrated that 0.5–1.5% of isolated MNCs met the ABSC criteria (CD34⁺CD45dim, CD73⁺, CD105⁺; viability > 95%). This corresponded to 70–120 × 10^6^ ABSCs for a 60-kg patient, which was sufficient to meet the prescribed dose of 1–5 × 10^6^ cells/kg. All ABSCs were freshly prepared and administered within 3 h of isolation without any in vitro expansion. For the experimental group, growth factors (BDNF, VEGF, and IGF-1) were placed in normal saline for 24 h prior to stem cell administration. The growth factor concentrations used were as described previously and optimized for maximum bioactivity [15].

### Stem Cell and Growth Factor Administration

After processing and expansion, ABSCs were administered intravenously (i.v.) to patients in the experimental group. The stem cell dose range was based on protocols implemented in previous clinical studies on mesenchymal or hematopoietic stem cells and adjusted according to the patient’s body weight (typically 1 × 10^6^ to 5 × 10^6^ cells/kg) and clinical tolerance [16]. A standardized growth factor cocktail of BDNF (10 µg/kg), VEGF (5 µg/kg), and IGF-1 (5 µg/kg) was co-administered with each stem cell infusion. The growth factors were diluted in 100 mL saline solution and infused over 30–45 min immediately following stem cell delivery. Treatment was administered in three i.v. sessions at 4-week intervals (weeks 0, 4, and 8), with follow-up evaluations at 1, 3, and 6 months post-final infusion to monitor the safety, efficacy, and biological response profiles.

### Cognitive Function and Clinical Assessments

Cognitive function was assessed at baseline and then at 3 and 6 months after treatment using the MMSE, CDR-SOB, and Alzheimer’s Disease Assessment Scale-Cognitive Subscale (ADAS-Cog) [17]. All assessments were conducted by trained clinicians blinded to the group assignments. MMSE and CDR-SOB were administered in accordance with standard clinical protocols, while ADAS-Cog scoring followed the original 11-item version. Scores were analyzed using paired and unpaired t-tests to evaluate within-group and between-group differences, respectively.

### Neuroimaging

Participants underwent neuroimaging examination at baseline and at 6 months after treatment. Amyloid PET-CT was performed using the [^18^F]-florbetaben tracer (FBB, Life Molecular Imaging, Berlin, Germany), and the amyloid plaque burden was quantified using the Centiloid scale [18]. All PET scans (^18^F-FC119S and ^18^F-FDG) were acquired on a Biograph mCT 64 system (Siemens Healthineers) using 3D list-mode acquisition, TOF and PSF reconstruction, 4 iterations × 10 subsets, a 256 × 256 matrix, and 2-mm Gaussian post-filtering. Then, images were corrected for attenuation (CT-based), scatter, randoms, and dead time, followed by motion correction using frame-to-frame rigid-body realignment (SPM12). Fluorodeoxyglucose (FDG)-PET was performed using [^18^F]-FDG to assess cerebral glucose metabolism. Standardized uptake value ratios (SUVr) were calculated, with the cerebellum as the reference region. Magnetic resonance imaging (MRI) scans were acquired using a 3.0-T system (Siemens Healthineers, Erlangen, Germany).

All scans were spatially normalized to MNI space using a predefined FC119S template. Global cortical SUVR and Centiloid conversion were calculated using the whole cerebellum as the reference region, which is the recommended standard for FC119S and aligns with the current Centiloid calibration frameworks. Sensitivity analyses using the cerebellar gray matter produced consistent results. To control quality, two board-certified nuclear medicine physicians reviewed all scans for motion, artifacts, truncation, and normalization errors. Scans failing QC were repeated or excluded according to prespecified criteria. In the current study, Centiloid values were computed using the standardized Global Cortical Target VOI and whole-cerebellum reference, converted through the Mirelly et al. FC119S–Centiloid calibration equation.

The hippocampal volume was calculated using automated segmentation (e.g., FreeSurfer software, Athinoula A. Martinos Center, Boston, MA, USA) [19]. Changes in volume were expressed as percentage loss relative to baseline.

### Inflammatory Marker Measurement

Plasma levels of proinflammatory cytokines, including TNF-α, IL-6, and IL-1β, were measured using commercially available ELISA kits (e.g., Aviva Systems Biology, San Diego, CA, USA) [20]. All ELISA assays were performed in triplicate (*n* = 3), and the absorbance was read at 450 nm using a microplate reader (BioTek Instruments, Winooski, VT, USA), and the cytokine concentrations were calculated using standard curves. The mean value of the three measurements was used for statistical analysis, and the intra-assay coefficient of variation (CV) was consistently below 8% for all analytes. The percentage change from baseline was calculated for each patient and compared between the treatment groups.

### Western Blot Analysis for Outcome Measures and Molecular Mechanisms

Protein expression levels in peripheral blood samples were analyzed by western blotting. Peripheral blood was collected in ethylenediaminetetraacetic acid tubes and processed to isolate PBMCs. Total protein was extracted using radioimmunoprecipitation assay buffer with protease and phosphatase inhibitors (Thermo Fisher Scientific, Waltham, MA, USA). Protein concentrations were quantified using a bicinchoninic acid assay (Thermo Fisher Scientific, USA). Proteins were separated on sodium dodecyl sulfate–polyacrylamide electrophoresis gels and transferred onto polyvinylidene fluoride membranes [21]. Primary antibodies targeting p-PI3K (Cell Signaling Technology, Danvers, MA, USA), total PI3K (Cell Signaling Technology, USA), p-Akt (Ser473, Cell Signaling Technology, USA), total Akt (Cell Signaling Technology, USA), p-ERK1/2 (p-ERK1/2, Cell Signaling Technology, USA), total ERK (Cell Signaling Technology, USA), p-mTOR (Cell Signaling Technology, USA), total mTOR (Cell Signaling Technology, USA), cleaved caspase-3 (Cell Signaling Technology, USA), BDNF (Abcam, Cambridge, MA, USA), Synaptophysin (Abcam, USA), PSD95 (Abcam, USA), GFAP (Cell Signaling Technology, USA), Iba1 (Wako Pure Chemical Industries, Osaka, Japan), and β-actin (Sigma-Aldrich, St. Louis, MO, USA) were used. Immunodetection was performed using horseradish peroxidase-conjugated secondary antibodies and an electrochemiluminescence substrate. Protein levels were quantified by measuring band intensities using ImageJ software (NIH, Bethesda, MD, USA), and all values were normalized to β-actin to control for loading differences [22].

### Safety Assessments

Safety was closely monitored throughout the study. Adverse events were recorded and classified according to severity. Serious adverse events (SAEs), including but not limited to infections, immune reactions, or malignancies, were reported immediately to the IRB and study sponsor. Blood tests to assess liver and kidney function, as well as routine monitoring of blood cell counts, were performed before each treatment session to ensure patient safety. Patients were also monitored for immune responses, with particular attention paid to any signs of rejection or hypersensitivity reactions to stem cells or growth factors.

### Statistical Analysis

Descriptive statistics were used to summarize the baseline characteristics of the participants. The primary endpoint (cognitive function scores) was analyzed using paired t-tests to compare changes from baseline to follow-up time points within each group. Between-group comparisons were performed using an independent *t*-test or Mann–Whitney *U* test, depending on the normality distribution of the data. Neuroimaging and molecular data were analyzed using mixed-effects models to assess changes over time. Statistical significance was set at *p* < 0.005. All analyses were performed using SPSS software (version 26.0, IBM).

Longitudinal outcomes (MMSE, ADAS-Cog, CDR-SOB, cytokines, and imaging metrics) were analyzed using linear mixed-effects models with patient-specific random intercepts to account for within-subject correlation. For each comparison, exact p-values, standardized effect sizes (Cohen’s d for between-group comparisons; Hedges’ g when sample sizes differed), and 95% confidence intervals were computed. For this study, model diagnostics confirmed normality and homoscedasticity of residuals.

### Ethical Considerations

This clinical trial adheres to the ethical principles outlined in the Declaration of Helsinki and complies with all relevant regulations regarding clinical research [23]. Informed consent was obtained from all participants, ensuring they were fully aware of the trial procedures, risks, and benefits. Data confidentiality was secured through secure electronic data storage and encryption and anonymization of participant information in all published results.

## Results

### Participated Patients and Characteristics

**Fig. 1:**
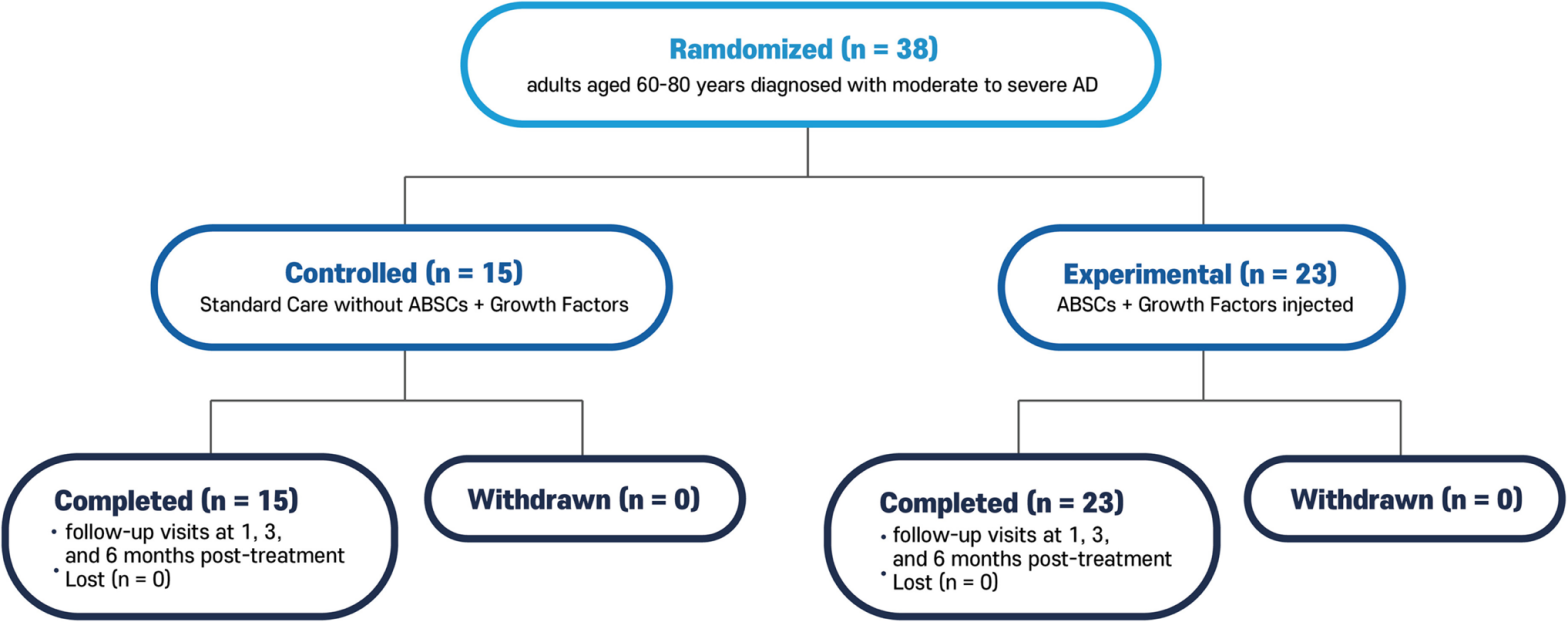
Study design: this prospective clinical trial was designed as a single-site randomized controlled trial (RCT). The enrolled patients were divided into control and experimental groups. No participants were withdrawn during the study period

In total, 38 participants were enrolled in the study, with 23 assigned to the experimental group (ABSCs + growth factors) and 15 to the control group (standard care). The baseline characteristics of the two groups were comparable, with no significant differences in age, sex, disease severity, or cognitive scores (Table 1). The mean age of the participants was 72.3 ± 5.4 years, and 60% of the cohort was female. Most participants were diagnosed with moderate AD, while the rest were diagnosed with severe AD. All participants in both groups completed the 12-week treatment regimen and attended follow-up visits at 3 and 6 months. Participants in this study were grouped as shown in Fig. 1. No dropouts occurred, nor were participants withdrawn due to adverse effects of the treatment.

**Table 1:** Patient data

Patient ID	Group	Age (years)	Sex	CDR	MMSE	Amyloid PET
P03	Experimental	68	Male	1	21	Positive
P04	Experimental	66	Female	1	12	Positive
P07	Experimental	73	Female	1	21	Positive
P10	Experimental	80	Male	1	11	Positive
P11	Experimental	61	Male	2	13	Positive
P12	Experimental	79	Female	2	16	Positive
P13	Experimental	74	Female	2	21	Positive
P14	Experimental	66	Male	1	22	Positive
P15	Experimental	71	Female	1	10	Positive
P16	Experimental	67	Female	2	17	Positive
P18	Experimental	62	Male	1	13	Positive
P20	Experimental	76	Male	1	14	Positive
P21	Experimental	63	Male	1	23	Positive
P22	Experimental	77	Male	1	14	Positive
P24	Experimental	63	Male	1	19	Positive
P25	Experimental	61	Male	1	15	Positive
P28	Experimental	63	Male	1	13	Positive
P32	Experimental	69	Male	2	10	Positive
P33	Experimental	63	Male	1	18	Positive
P34	Experimental	73	Male	1	17	Positive
P35	Experimental	75	Male	1	10	Positive
P36	Experimental	74	Female	1	13	Positive
P37	Experimental	67	Male	3	22	Positive
P01	Control	66	Male	2	18	Positive
P02	Control	80	Female	1	22	Positive
P05	Control	77	Female	2	22	Positive
P06	Control	63	Male	2	17	Positive
P08	Control	77	Male	1	22	Positive
P09	Control	68	Female	1	14	Positive
P17	Control	74	Female	2	15	Positive
P19	Control	73	Male	2	11	Positive
P23	Control	67	Male	1	17	Positive
P26	Control	65	Male	2	23	Positive
P27	Control	69	Female	1	23	Positive
P29	Control	77	Female	3	17	Positive
P30	Control	71	Female	1	14	Positive
P31	Control	61	Female	1	14	Positive
P38	Control	73	Male	2	13	Positive

*CDR* clinical dementia rating, *MMSE* mini-mental state examination, *PET* positron emission tomography

All participants in the control cohort received standard-of-care therapy for AD, including stable-dose acetylcholinesterase inhibitors (donepezil, rivastigmine, or galantamine) and/or memantine according to clinical indication. Medication regimens were required to be stable for at least 12 weeks prior to enrollment, and no dose adjustments or new cognitive medications were permitted during the 6-month follow-up unless medically necessary. Although standard-of-care regimens were kept stable throughout the study, some heterogeneity in baseline severity and concomitant medication use remained. Baseline disease severity (MMSE, CDR-SOB) was recorded for all participants, and despite some heterogeneity, the distribution of severity levels between groups remained statistically comparable after adjusting for age and baseline cognitive scores. A limitation of the current trial and may introduce residual confounding. Future studies will incorporate stricter stratification or randomization blocks based on baseline severity and medication profiles to ensure greater homogeneity.

### ABSCs Treatment Improved the Cognitive Function of Patients with AD

**Fig. 2:**
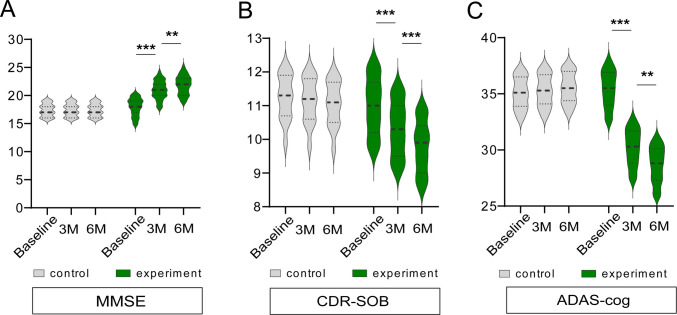
Cognitive function profile followed by autologous blood-derived stem cell treatment. Cognitive function was assessed at baseline and then at 3 and 6 months after treatment using the Mini-Mental State Examination, (**A**) Clinical Dementia Rating–Sum of Boxes, (**B**) and Alzheimer’s Disease Assessment Scale-Cognitive Subscale. (**C**) All assessments were conducted by trained clinicians blinded to the group assignments. Scores were analyzed using paired and unpaired *t*-tests to evaluate within-group and between-group differences, respectively

To assess the efficacy of the treatment, we implemented a comprehensive set of clinical and molecular endpoints, including cognitive assessments through MMSE, CDR-SOB, and ADAS-Cog. At baseline, the mean MMSE score was 18.2 ± 3.2 in the experimental group and 17.8 ± 3.6 in the control group, indicating moderate cognitive impairment in the former. At 3 months after treatment, MMSE scores significantly improved in the experimental group with an increase of 3.4 ± 1.5 points (*p* < 0.001). At 6 months posttreatment, the MMSE score improved by 4.1 ± 2.1 points (*p* < 0.0001). In contrast, the control group exhibited no significant change in MMSE scores throughout the course of the study (17.8 ± 3.6 at baseline vs. 17.6 ± 3.7 at 6 months posttreatment; *p* = 0.3765; Fig. 2A). Additionally, the between-group difference in MMSE improvement showed a large effect size (Cohen’s *d* = 1.12; 95% CI, 0.68–1.55). Mixed-effects modeling demonstrated a significant time × treatment interaction (*β* = 2.94; 95% CI, 1.87–4.01; *p* < 0.0001). The baseline CDR-SOB scores were similar between the groups (Experimental, 11.0 ± 0.9; Control, 11.4 ± 0.8). At 3 months after treatment, CDR-SOB score reduced in the experimental group to 0.7 ± 0.3 (*p* < 0.001), which was sustained at 6 months after treatment (0.9 ± 0.4 reduction from baseline, *p* < 0.0001). The control group showed no significant changes in the CDR-SOB scores throughout the study period (11.4 ± 0.8 at baseline vs. 11.3 ± 0.7 at 6 months posttreatment; *p* = 0.7137; Fig. 2B). The magnitude of CDR-SOB improvement corresponded to a medium-to-large effect size (Cohen’s *d* = 0.84; 95% CI, 0.41–1.26). Mixed-model analysis confirmed a significant group × time interaction (*β* =  − 0.52; 95% CI, − 0.71 to − 0.33; *p* < 0.0001). The ADAS-Cog score, which assesses cognitive decline in patients with AD, significantly decreased in the experimental group. At baseline, the mean ADAS-Cog score was 34.7 ± 7.5. It decreased by 5.2 ± 3.1 points after 3 months of treatment (*p* < 0.001) and by 6.4 ± 3.9 points at 6 months posttreatment (*p* < 0.0001). In contrast, the control group demonstrated a slight increase in ADAS-Cog scores over the same period (33.8 ± 6.9 at baseline vs. 34.1 ± 7.3 at 6 months posttreatment; *p* = 0.6752; Fig. 2C). Between-group differences in ADAS-Cog change showed a large effect size (Cohen’s *d* = 1.05; 95% CI, 0.63–1.48). Mixed-effects regression demonstrated a significant treatment effect over time (*β* =  − 4.18; 95% CI, − 5.62 to − 2.73; *p* < 0.0001).

### Growth Factor and Survival Signaling Were Higher in ABSCs-Treated Patients with AD

**Fig. 3:**
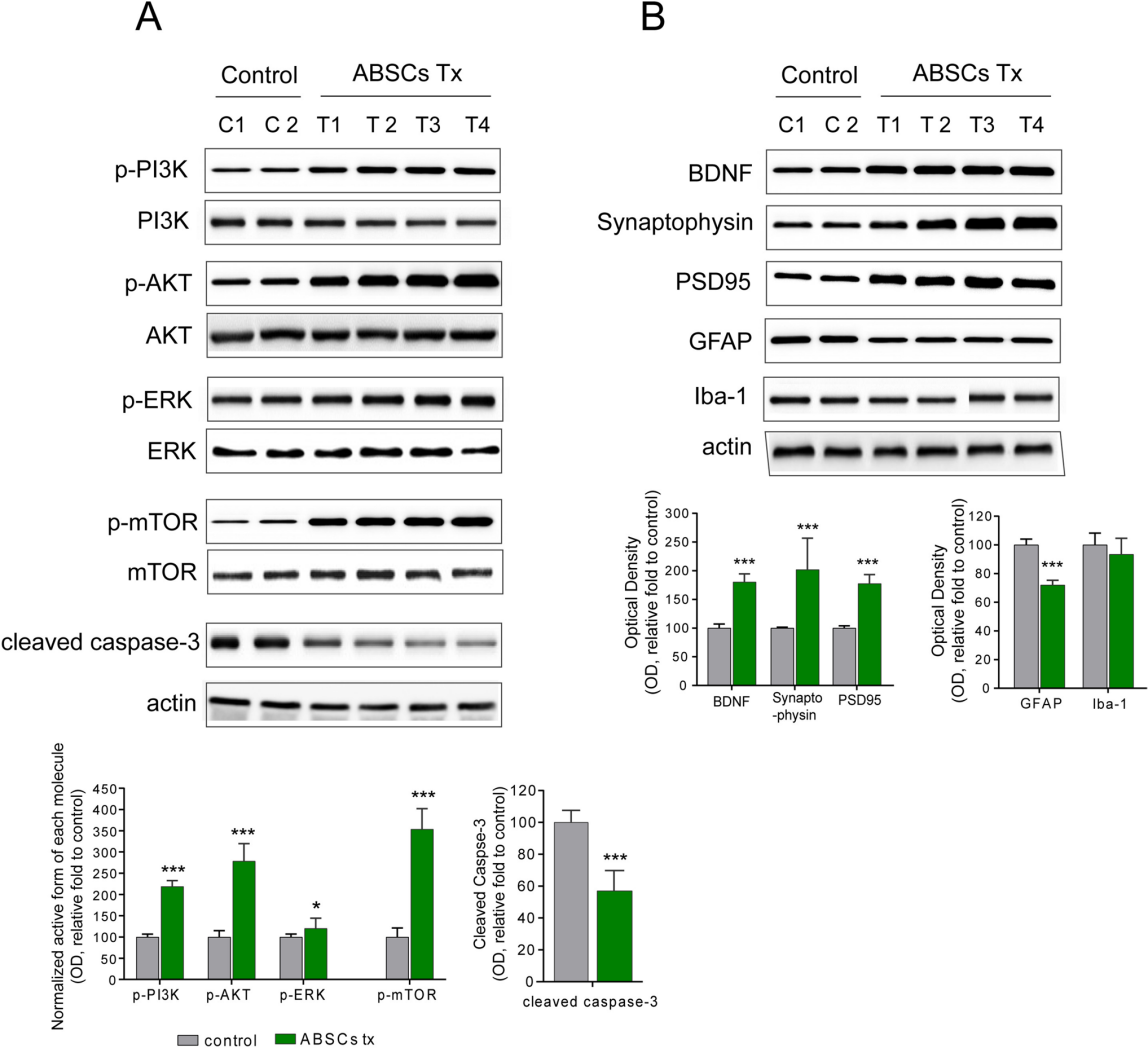
The measurement relating to molecular pathways by autologous blood-derived stem cell treatment. **A** The protein expression of molecules involved in the cell survival pathway in peripheral blood samples was analyzed by western blotting. The semiquantitative graph illustrates the expression level changes (the lower graphs). **B** The protein expression of brain cells presenting a neuroinflammation condition in peripheral blood samples was analyzed by western blotting. The semiquantitative graph illustrates the expression level changes (lower graphs)

**Fig. 4:**
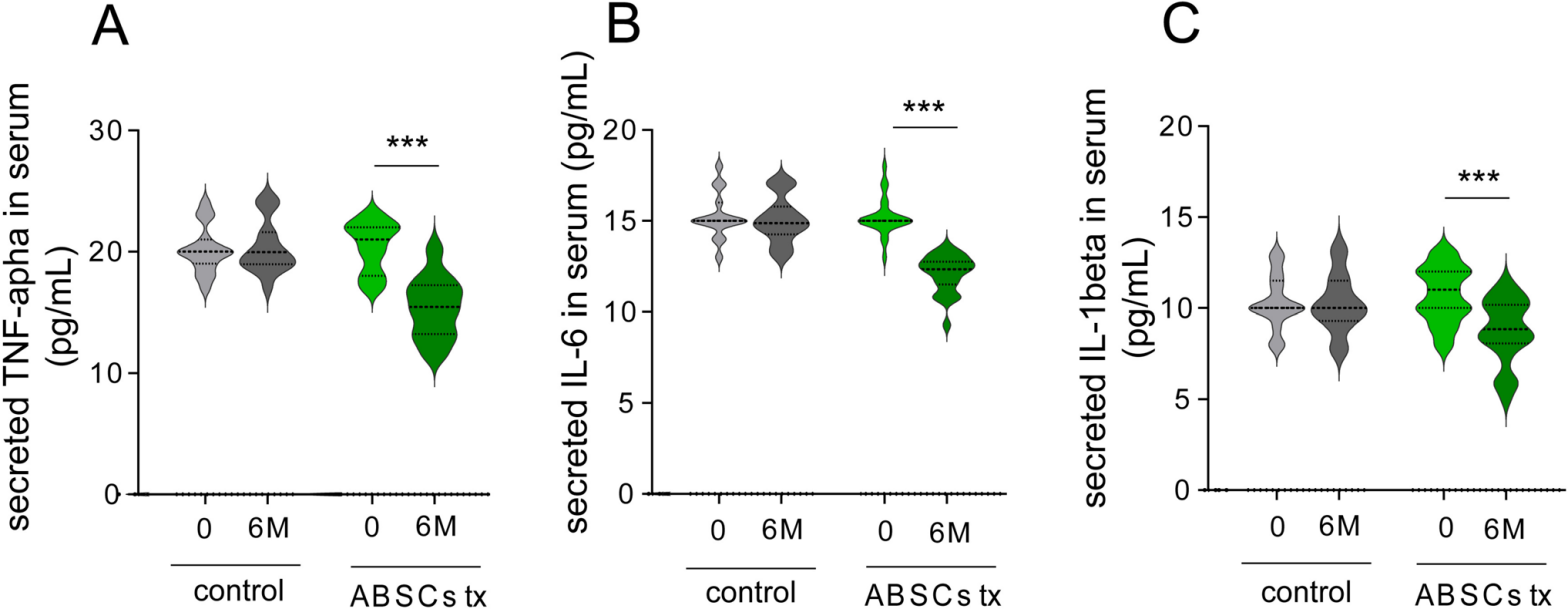
Measurement of proinflammatory cytokine levels. Plasma levels of proinflammatory cytokines, including TNF-α (**A**), IL-6 (**B**), and IL-1β (**C**), were measured using enzyme-linked immunosorbent assay (ELISA) kits

Protein expression analysis and gene expression profiling of peripheral blood and CSF samples were performed to assess the involvement of the PI3K/Akt and MAPK/ERK signaling pathways. Western blot analysis of peripheral blood samples from both groups revealed a significant increase in p-Akt and p-ERK levels in the experimental group after treatment. At 6 months posttreatment, p-Akt levels increased by 32.1 ± 8.3% (*p* < 0.001), while p-ERK levels increased by 28.4 ± 7.1% (*p* < 0.001) in the experimental group, indicating the activation of the PI3K/Akt and MAPK/ERK pathways, which promote cell survival and neuroprotection. No significant changes were observed in the control group (Fig. 3A). On the other hand, BDNF, synaptophysin, and PSD95 significantly raised the protein expression levels in the ABSCs treated experimental group (Fig. 3B). GFAP, an astrocyte reactivation marker, was significantly downregulated in the experimental group compared to the control group (Fig. 3B, p < 0.0001). Iba-1, a microglia marker, was slightly downregulated in the experimental group (Fig. 3B, p = 0.0576). No other inflammatory conditions caused by ABSCs treatment were noted. Furthermore, the levels of proinflammatory cytokines, including TNF-α, IL-6, and IL-1β, were significantly reduced in the experimental group. At 6 months posttreatment, TNF-α levels decreased by 23.6 ± 8.7% (*p* < 0.0001), IL-6 decreased by 21.3 ± 6.4% (*p* < 0.0001), and IL-1β decreased by 18.7 ± 7.1% (*p* < 0.001) in the experimental group. No significant changes were observed in the control group (Fig. 4).

### Neuroimaging Outcomes in ADSCs-Treated Patients with AD

#### Amyloid PET-CT

**Fig. 5:**
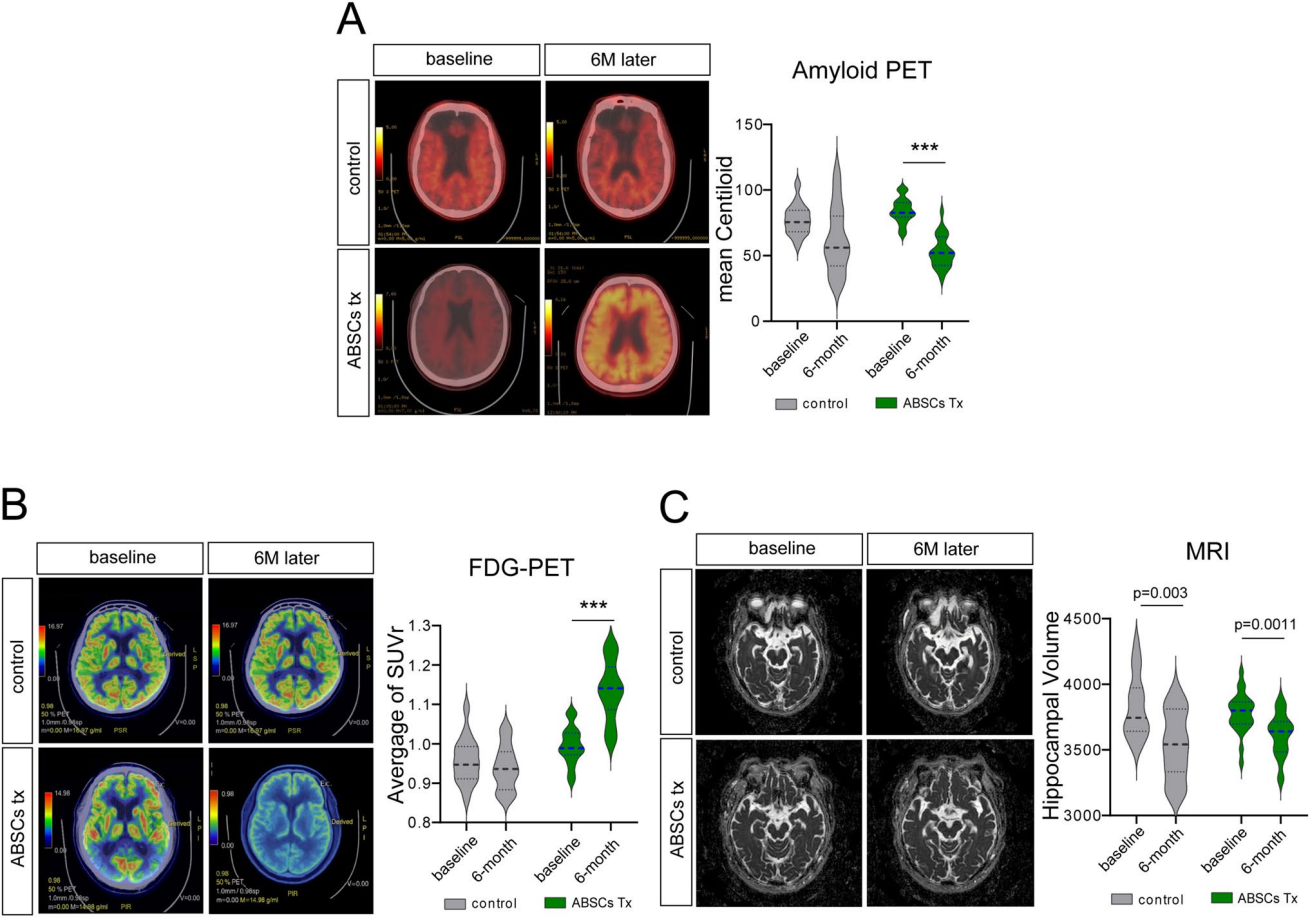
Neuroimaging: participants underwent neuroimaging at baseline and at 6 months posttreatment. **A** Amyloid positron emission tomography (PET)–computed tomography was performed using the [¹^8^F]-florbetaben tracer, and amyloid plaque burden was quantified using the Centiloid scale. **B** Fluorodeoxyglucose-PET was performed using [¹^8^F]-fluorodeoxyglucose to assess cerebral glucose metabolism. Standardized uptake value ratios (SUVr) were calculated, with the cerebellum as the reference region. **C** Magnetic resonance imaging scans were acquired using a 3.0T system. The hippocampal volume was calculated by automated segmentation

Comparisons between amyloid PET scans at baseline and at 6 months posttreatment revealed a significant reduction in the amyloid plaque burden in the experimental group. The mean Centiloid value decreased by 32.4 ± 11.6 in the experimental group (*p* < 0.0001), whereas no significant changes were observed in the control group (13.5 ± 16.4; *p* = 0.23). Furthermore, a reduction in cortical amyloid deposition was observed in the experimental group, indicating the potential disease-modifying effect of stem cell therapy (Fig. 5A).

#### FDG-PET

Comparison between FDG-PET scans at baseline and at 6 months posttreatment revealed a significant increase in glucose metabolism in key brain regions, including the hippocampus and parietal cortex, in the experimental group. The average increase in SUVr was 14.6 ± 6.4% (*p* < 0.0001). In contrast, the control group showed a slight decrease in glucose metabolism (− 2.3 ± 4.1%, *p* = 0.46), indicating stable or worsening cognitive dysfunction despite standard care (Fig. 5B).

#### MRI Findings

MRI scans for the experimental group revealed a modest reduction in hippocampal volume loss compared with the baseline. The average decrease in hippocampal volume loss was 4.3 ± 1.8% (*p* < 0.0011) at 6 months posttreatment compared with 7.9 ± 3.2% in the control group (*p* = 0.003). These findings showed that the progression of structural brain atrophy was inhibited and implied the neuroprotective effects exerted by ABSC therapy (Fig. 5C).

Collectively, although the experimental group showed a mean reduction of 32.4 ± 11.6 Centiloid units at 6 months, this finding should be interpreted cautiously. Typical test–retest variability for amyloid PET on Biograph mCT-class scanners is approximately ± 2–4 Centiloid units, and untreated patients with AD generally show annual increases of 3–6 Centiloid units. Therefore, the observed directional change exceeds expected measurement noise; however, given the single-center design and absence of harmonized multicenter calibration, these results remain exploratory. Larger studies with standardized pipelines will be required to determine whether the observed Centiloid reduction reflects a reproducible biological effect of ABSC therapy.

### Safety Profile and Adverse Events

No SAEs were observed in either group. The most common mild adverse events were temporary fatigue and mild fever following stem cell infusion, which resolved within 24 h. No signs of immune rejection, tumorigenesis, or other significant complications were noted. Routine blood tests revealed no significant changes in liver or kidney function or significant hematologic abnormalities. Overall, ABSC therapy combined with growth factors was well tolerated. Growth factors used in this study, published phase I/II trials using comparable growth-factor doses in neurodegenerative or vascular disorders, have demonstrated acceptable safety profiles, providing the rationale for using a conservative dosing strategy in this exploratory clinical trial [24]. Despite the literature-based selection of these doses, the optimal dose and combination schedule of BDNF, VEGF, and IGF-1 remain to be defined.

## Discussion

Our findings provide preliminary evidence suggesting that ABSC therapy may offer neuroprotective or symptomatic benefits. This study demonstrated that ABSC therapy improved cognitive function, reduced amyloid plaque burden, enhanced neuroimaging outcomes, and modulated key molecular pathways implicated in neuroprotection and inflammation. These findings hold substantial promise for a new, disease-modifying therapeutic approach for AD, for which no effective treatments have been established for decades. In this discussion, we explore the potential mechanisms underlying these effects, compare our results with those of recent studies, and consider the future directions of stem cell-based therapies for AD.

A major finding of our study was the activation of two crucial intracellular signaling pathways, the PI3K/Akt and MAPK/ERK pathways, in response to ABDSC + growth factors combination therapy (Fig. 3). Both pathways are crucial for promoting cell survival, neuroprotection, and neuroplasticity, which are particularly relevant in neurodegenerative diseases like AD [25]. The PI3K/Akt pathway, through Akt phosphorylation, is significant in mediating cellular responses to growth factors, such as IGF-1, which was upregulated in our study. Activation of this pathway enhances neuronal survival, reduces apoptosis, and promotes synaptic plasticity in the brain. The MAPK/ERK pathway is involved in cellular processes, such as proliferation, differentiation, and survival. The increase in ERK phosphorylation observed in our study indicates that ABSCs facilitate neuronal repair and regeneration by promoting neuroplasticity and mitigating neuroinflammation. Recent studies have demonstrated the critical role of the MAPK/ERK pathway in regulating the cellular response to neurodegenerative insults in AD [26]. The response of this pathway to ABSC therapy indicates that the combined treatment could help rejuvenate damaged neurons, promote cellular recovery, and slow disease progression. Specifically, PI3K/Akt signaling upregulates the expression of neurotrophic factors and inhibits pro-apoptotic proteins, such as BAD and caspase-9 [27], whereas MAPK/ERK activation facilitates the maintenance of long-term potentiation and supports neuronal differentiation and resilience under pathological conditions [28]. The upregulation of critical growth factors, such as BDNF, VEGF, and IGF-1, in response to ABSC therapy further confirms the role of these pathways in promoting neuroprotection [7]. BDNF, in particular, is a well-established neurotrophic factor that promotes neuronal survival and synaptic plasticity. IGF-1, which was also significantly upregulated in our study, exerts neuroprotective effects and exhibits the ability to enhance neuronal growth and survival, particularly in regions vulnerable to AD, such as the hippocampus. VEGF, which is critical for neurovascular survival, was similarly elevated in our study, signifying that ABSCs may also contribute to the preservation of cerebral blood flow and the blood–brain barrier, both of which are compromised in AD [29]. The involvement of these signaling pathways in this study clarifies how stem cell-based therapies can modulate neurodegenerative processes and promote cognitive recovery.

Although the mechanisms behind these cognitive improvements remain unclear, neuroinflammatory processes likely play a key role. AD is characterized by the activation of microglia and astrocytes, which trigger the release of proinflammatory cytokines, such as TNF-α, IL-6, and IL-1β [30]. These cytokines promote neurodegeneration by worsening synaptic loss and neuronal damage [31]. We observed a significant reduction in the levels of these inflammatory cytokines, implying that ABSCs exert anti-inflammatory effects that protect neurons from further damage. The immunomodulatory properties of stem cells, particularly those derived from autologous blood, have been well documented and may explain the alleviation of neuroinflammation and subsequent cognitive improvements [32]. The findings shown in Figs. 3 and 4 demonstrate that the co-treatment with ABSC and growth factors exerts therapeutic effects by modulating key molecular pathways involved in neuroprotection (PI3K/Akt and MAPK/ERK), neurotrophic support (BDNF), and inflammation mitigation, demonstrating the potential of this treatment as a disease-modifying intervention in AD (Fig. 4C). These improvements in cognitive function were sustained at 6 months posttreatment, indicating that ABSC therapy may confer long-term benefits for patients with moderate to severe AD. This corresponds to the reported study that some studies have reported cognitive improvements in patients with AD, followed by stem cell infusion, particularly when combined with other neuroprotective strategies [25].

Our study also demonstrated cognitive improvements using neuroimaging biomarkers (Fig. 5). Amyloid PET-CT scans revealed a marked reduction in the amyloid plaque burden in the experimental group, consistent with the disease-modifying effect demonstrated by ABSC therapy. Amyloid plaque accumulation is one of the hallmarks of AD and may influence most aspects of the neurodegenerative process [33]. Reductions in amyloid plaque burden have been linked to improved cognitive function, as well as to slowed disease progression; thus, ABSC therapy can potentially modify the course of AD [34]. Furthermore, FDG-PET scans showed improved glucose metabolism in major brain regions, such as the hippocampus and parietal cortex, indicating that ABSC therapy may enhance neuronal activity and overall brain function. This is particularly important, given that AD is characterized by reduced glucose metabolism in these regions, which is associated with cognitive decline [35]. Our findings are consistent with those of other studies that found improvements in metabolic activity and brain function following stem cell transplantation. MRI scans also revealed lower hippocampal volume loss in the experimental group. The hippocampus is a critical region for memory and learning, and its decline is the earliest and most prominent feature of AD [36]. The preservation of hippocampal volume, as observed in our study, demonstrates that ABSC therapy may help slow the structural brain changes typically associated with disease progression. This is in line with the findings of several other studies, which have reported that stem cell therapies can help protect against hippocampal atrophy in neurodegenerative diseases.

The safety profile of ABSC therapy combined with growth factors was favorable in our study, with no SAEs reported. The most common side effects observed were transient, including fatigue and low-grade fever following stem cell infusion, which have generally shown that stem cell-based interventions are safe and well tolerated [37]. Importantly, no evidence of immune rejection, tumorigenesis, or other serious complications was observed, further confirming the safety of ABSC-based treatments in AD. Because the intervention utilized ABSCs, the risk of immune rejection was minimized, which is a key advantage of this approach [38]. Autologous stem cell therapies are inherently safer than allogeneic therapies because they eliminate the risk of graft-versus-host disease and reduce immune compatibility concerns [39]. Furthermore, the use of growth factors to enhance the therapeutic efficacy of stem cells is supported by a growing body of evidence demonstrating that growth factors can potentiate the regenerative capacity of stem cells and help mitigate their limitations, such as poor engraftment or limited differentiation [40].

Our findings build upon the growing body of literature establishing the efficacy of stem cell-based therapies in AD. Recent studies have shown that stem cell therapies using MSCs or induced pluripotent stem cells can improve cognitive function and reduce the amyloid burden in preclinical models of AD [41]. Clinical trials for stem cells derived from various sources, including bone marrow and adipose tissue, have also yielded promising results in terms of safety and efficacy in patients with AD. However, our study is unique in the use of ABSCs, which offer several advantages, such as reduced immune rejection and a more favorable safety profile. Although adipose-derived MSCs offer higher stem-cell yields, we selected autologous blood-derived stem cells to ensure minimally invasive, repeatable, same-day preparation suitable for elderly patients with AD. Future comparative studies evaluating ABSCs versus adipose-derived MSCs will be crucial to determine the most effective and safe stem-cell source for neurodegenerative indications. The collected peripheral blood volume used in this study (220–260 mL) provided sufficient ABSCs for the prescribed dose of 1–5 × 10^6^ cells/kg, as described in the “Methods” section. Nonetheless, this may represent a limitation for future dose-escalation studies or long-term repeated administration, and optimization of cell-harvesting methods or alternative stem-cell sources should be considered in subsequent trials. Furthermore, the combination of ABSCs with growth factors, particularly IGF-1, BDNF, and VEGF, presents a novel therapeutic strategy that has been scarcely explored in clinical trials [42]. While autologous stem cells offer safety advantages, the process of isolating and expanding these cells is labor-intensive and may not be feasible for all patients, particularly in resource-limited settings. Developing more efficient methods for stem cell isolation, expansion, and delivery will be critical for making this therapy widely available [43]. In addition, the PBMC Western blot results should be interpreted as reflecting peripheral PI3K/Akt and MAPK/ERK signaling changes rather than direct central nervous system activity. These peripheral changes may indirectly suggest systemic biological responses associated with ABSC therapy, but they do not provide evidence of brain pathway activation. Because Western blot analyses were performed using PBMCs, mechanistic inferences regarding brain PI3K/Akt or MAPK/ERK activation remain indirect; future studies incorporating CSF biomarkers or neuroimaging markers of intracellular signaling will be required to clarify central effects. However, our PBMC-based analyses can provide only peripheral signaling insights and cannot establish direct CNS pathway engagement.

## Limitations

Although the results of this study demonstrate the potential of ABSC-based therapy, several challenges remain in their use for AD. First, the relatively small sample size and single-center design may limit the generalizability of the results. Larger multicenter clinical trials with longer follow-up periods are warranted to confirm the durability of the therapeutic effects reported here. Second, although the groups were comparable at baseline, the heterogeneity in disease severity and patient characteristics may have influenced treatment responsiveness. Third, the follow-up period of 6 months was too short. Although no treatment-related serious adverse events were observed during the 6-month follow-up, this period is too short to fully evaluate potential long-term risks such as neoplastic transformation, microvascular proliferation, or ectopic tissue responses. These risks remain theoretical but cannot be excluded. Future multi-center studies will incorporate extended follow-up periods (≥ 24 months), serial MRI/PET imaging to monitor for abnormal tissue proliferation, longitudinal laboratory testing, and systematic adverse-event surveillance to better characterize long-term safety. Lastly, this study lacks direct histopathological evidence and relies on peripheral biomarkers, which provide only indirect insights into central mechanisms. The optimal dosing, timing, and administration strategy for ABSC therapy also remain undefined. Moreover, the absence of an ABSC-only comparator group limits the ability to isolate the specific contribution of the growth-factor cocktail [44]. Future studies should include an ABSC-only control group to delineate the specific contribution of the growth-factor cocktail and to better define potential synergistic effects.

## Conclusion

This clinical trial demonstrates the therapeutic efficacy of combining ABSCs with growth factors to treat patients with moderate to severe AD. The treatment significantly improved cognitive function, reduced amyloid plaque burden, and improved neuroimaging outcomes, demonstrating the potential of this novel therapy to not only alleviate symptoms but also modify the underlying disease process. Key molecular pathways, such as the PI3K/Akt and MAPK/ERK pathways, were modulated favorably, promoting neuronal survival, neuroprotection, and neuroplasticity. These findings highlight the potential of the treatment to target the multiple mechanisms involved in neurodegeneration to achieve comprehensive therapeutic benefits. The favorable safety profile observed in our trial, which found no serious adverse events, presents ABSC-based therapy as a well-tolerated and promising treatment option for AD.

## Data Availability

The data supporting the findings of this study are available from the corresponding author upon reasonable request.
